# Early de-cannulation from extracorporeal membrane oxygenation following ventricular tachycardia radiofrequency ablation

**DOI:** 10.3389/fcvm.2022.998079

**Published:** 2022-10-18

**Authors:** Avi Sabbag, Johnatan Nissan, Roy Beinart, Leonid Sternik, Igal Kassif, Alexander Kogan, Eilon Ram, Eyal Nof

**Affiliations:** ^1^Davidai Arrhythmia Center, Sheba Medical Center, Ramat Gan, Israel; ^2^Sackler Faculty of Medicine, Tel Aviv University, Tel Aviv-Yafo, Israel; ^3^Department of Cardiac Surgery, Sheba Medical Center, Ramat Gan, Israel

**Keywords:** vetricular tachycardia, extracorporeal membrane oxygenation, ablation, circulatory support, early de-cannulation

## Abstract

**Objectives:**

Ventricular tachycardia ablation (VTA) with hemodynamic compromise presents a challenge. Veno-arterial extracorporeal membrane oxygenation (VA-ECMO) support allows the safe completion of the procedure. There are limited data regarding the safety of weaning off VA-ECMO at the end of the procedure. We report our experience with early VA-ECMO de-cannulation after VTA.

**Materials and methods:**

All patients undergoing VA-ECMO-assisted VTA, between January 2013 and December 2020 at a large tertiary center were included. Clinical characteristics, history of arrhythmia, procedural details, and outcomes were collected. Patients weaned from VA-ECMO immediately at the end of the procedure were compared to those that were de-cannulated at a later time.

**Results:**

A total of 46 patients (93.5% male, age 62 ± 10 years) were ablated with VA-ECMO support. Most had ischemic cardiomyopathy (65%) and (70%) presented with VT storm. The clinical VT was induced in the majority of patients (76%). A total of 99 VTs were induced of which 76 (77%) were targeted and successfully ablated. Non-inducibility was achieved in 74% of cases and most patients (83%) were de-cannulated at the end of the procedure on the procedure table. Survival at 1 year was higher among early de-cannulated patients (86 vs. 38% [log-rank *p*-value < 0.001]). At 1-year follow-up, 91.3% of surviving patients were free of appropriate ICD shocks.

**Conclusion:**

De-cannulation from VA-ECMO may be done immediately at the conclusion of VTA in most cases. Failure to timely wean off VA-ECMO is a strong predictor of mortality.

## Introduction

Ventricular tachycardia ablation (VTA) has been well established as an important and effective therapy for drug refractory arrhythmia in the context of structurally abnormal hearts (1). Yet, the procedure is challenging since 70% of VTs are not tolerated by the patients limiting the ability to carry out extensive activation or entrainment mapping (2). Furthermore, even when adopting a substrate-based approach one must nevertheless strive to achieve complete non-inducibility at the end of the ablation. This may result in the repeated induction of VTs until the desired endpoint is met. The combined effect of anesthesia, positive pressure ventilation, fluid overload, and reduced cardiac output during VTs may lead to acute decompensations requiring rescue circulatory support accompanied by high mortality risk (3).

The preemptive use of circulatory support in selected high-risk patients has been demonstrated to be both safe and effective (4). It allowed prolonged mapping during VT thus increasing the likelihood of achieving complete and successful ablation as well as preventing hemodynamic collapse (4). While several circulatory support systems have been explored with similar rates of success (5, 6), veno-arterial extracorporeal membrane oxygenation (VA-VA-ECMO) may offer hemodynamic support without limiting access to the LV and without inducing electromagnetic interference (7). The safety of using VA-ECMO to support VT in high-risk patients has been reported (7) but, to the extent of our knowledge, the feasibility of immediate de-cannulation at the end of the ablation has not been specifically evaluated. Therefore, we sought to determine the safety and feasibility of immediate de-cannulation, preemptive deployment of VA-ECMO support, at the end of the ablation procedure, a preemptive deployment of VA-ECMO support.

## Materials and methods

### Study design

**FIGURE 1:**
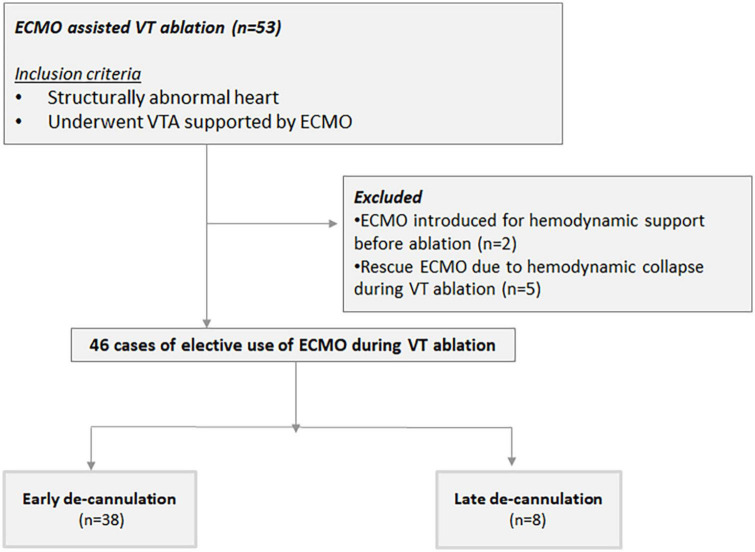
Flowchart summary from consent and eligibility for the study 1-year follow-up.

All cases of structurally abnormal heart that underwent VTA supported with a VA-ECMO at the Sheba Medical Center from January 2013 to December 2020 were reviewed and retrospectively analyzed. A structurally abnormal heart was defined as any cardiomyopathy, valvulopathy, or congenital heart disease impairing the ventricular function. Patient data, including demographics, and clinical and procedural features were obtained from our VTA registry. Only cases with a preemptive deployment of the system, done specifically to support the VTA procedure, were included. Preemptive VA-ECMO use was defined as the pre-planned deployment of the system in hemodynamically stable patients prior to the initiation of the VTA. Patients supported by VA-ECMO due to cardiogenic shock prior to the VTA or cases of unplanned and urgent introduction of VA-ECMO during VTA due to acute decompensation were excluded from the current analysis (Figure 1). The main study endpoints were a composite of VT, VF, or appropriate ICD shock as well as in-hospital all-cause mortality, defined as death occurring before discharge from the index hospitalization including transfer to other acute care facilities (8) and all-cause mortality. The study was approved by the local ethical board. Due to the retrospective nature of the analysis, no individual consent was required.

### Study population

Patients were referred for VTA in accordance with current guideline indications (1). Prior to the procedure, all patients underwent a clinical evaluation including the assessment of their functional status, an echocardiographic exam, and a review of arrhythmia history. The preemptive VA-ECMO support was planned when one or more of the following criteria were met: (1) severe aortic stenosis; (2) severe pulmonary hypertension; (3) severe (NYHA functional class III–IV) or decompensated heart failure; (4) documentation of hemodynamically unstable VT; and (5) previously failed VTA when the reason for failure was the instability of VT or patient during the procedure; and (6) Idiopathic dilated cardiomyopathy.

Patients were considered in VT storm if presented with three sustained ventricular arrhythmia requiring defibrillation. Decompensated heart failure was defined as acute worsening signs and symptoms of HF resulting in hospitalization. The diagnosis of severe AS required a mean trans-valvular gradient of ≥40 mmHg, a maximal velocity ≥ m/s, a calculated aortic valve area of ≤1 cm^2^, or a calcium score ≥ 2,000 in men and ≥1,500 in women (9). Pulmonary hypertension was defined as severe when systolic pulmonary artery pressure (sPAP) estimated by echocardiography was ≥60 mmHg or when right heart catheterization measured a mean pulmonary artery pressure (mPAP) ≥ 35 mmHg or mPAP ≥ 25 mmHg with low cardiac index (<2 L/min/m^2^) (10). Each patient’s eligibility criteria for ECMO-supported VTA are shown in a Supplementary Table.

### Extracorporeal membrane oxygenation

The VA-ECMO circuit setup included a centrifugal pump and a coated polymethylpentene oxygenator (Revolution [Sorin, Italy], Centrimag [Levitronix, Waltham, MA], and Rotaflow [Maquet, Germany]). The left femoral artery and vein were percutaneously cannulated in all patients. An outlet (23/25F) and inlet (15/17F) cannula were advanced to the right atrium and external iliac artery, respectively. The pump was set to a minimal baseline flow of 1.7–2 L/min. During VT or programmed stimulation, the flow was increased up to 3.5–4 L/min as needed to maintain hemodynamic stability and adequate systemic organ perfusion. A target ACT of 300 s was mandatory throughout the procedure.

### Ablation procedure

Electro-anatomical mapping was performed (CARTO 3 or XP, Biosense Webster, Diamond Bar, CA, USA) using a 3.5-mm tip open irrigated catheter (NaviStar ThermoCool, Biosense Webster, Diamond Bar Ca). Voltage maps were created during sinus rhythm. Peak-to-peak bipolar electrogram amplitude < 0.5 mV was defined as dense scar, voltage ≥ 0.5 and <1.5 mV as scar border zone (11). Epicardial mapping and ablation were carried out when deemed necessary.

All inducible sustained monomorphic ventricular tachycardia (SMVT) were targeted for ablation. Sites were targeted for ablation if pacing entrained the SMVT with concealed fusion and a post-pacing interval (PPI) within 30 ms of the VT cycle length (CL) or by activation mapping. In any case, scar substrate modification (“scar de-channeling”) was performed. Sites with low amplitude fractionated electrograms with stimulation to QRS, late potentials, or the best pace map sites were targeted. Pace mapping and entrainment mapping utilized unipolar stimuli with a strength of 10 mA and a pulse width of 2 ms. Radiofrequency (RF) energy was delivered at a power of 25–50 Watts targeting an impedance drop of at least 10 ohms. The endpoint of all procedures was non-inducibility of any VT with programmed stimulation (PS) at a basic drive train of 600 and 400 ms with up to three extrastimuli. A procedure was defined as successful if the patient was non-inducible for any VT following PS. Partial success was declared when only the clinical VT was no longer inducible and acute procedural failure was declared if the clinical VT was still inducible.

At the end of the ablation, the feasibility of immediate de-cannulation from ECMO in the electrophysiology lab was assessed based on the following criteria: (1) a mean atrial pressure ≥ 60 mmHg for at least 5 mins with minimal ECMO flow (<1 L/min) and minimal ionotropic support (<0.02 mcg/Kg/min of noradrenaline); (2) oxygen saturation > 92% on FiO_2_ ≤ 40%; (3) post-ablation lactate level < 30 mmg/dl; and (4) no severe acute complication, such as tamponade. Patients meeting all of these criteria were gradually weaned of circulatory support. Patients successfully weaned from ECMO before leaving the EP lab were defined as early de-cannulation (group A) and those that required additional prolonged support were considered late de-cannulation (group B).

Blood pressure was measured continuously and lactate levels were monitored carefully to identify any sign of decompensation and need for the reinstitution of circulatory support. If mean blood pressure > 60 was maintained on minimal ECMO flow (<1 L/min), we proceeded with de-cannulation. If the above-mentioned blood pressure could not be maintained in a patient, ECMO flow was increased and he was transferred to the intensive care unit (ICU) for another attempt. The decision to wean off ECMO in ICU was made at the discretion of the attending physician). Prior to VA-ECMO removal, protamine was administered with a target ACT of <180 s. After the removal of ECMO cannulas, manual pressure was applied to the groin. Immediate de-cannulation was defined as completing the entire weaning process before leaving the procedure room.

### Follow-up

Following the ablation, all patients were admitted to a monitored bed until discharged. Procedural and post-procedural complications were noted. All patients were seen at the device clinic 1 and 3 months after the procedure as well as continued biannual device examinations. The occurrence of VT and appropriate device therapies were collected. Mortality data were extracted from the Israeli National Population Register and were available for all cases. The date on heart transplantation or the implantation of a left ventricular assist device was collected from the national heart transplant register and was also available for all cases. The Institutional Review Board of Sheba Medical Center approved this registry on the basis of strict maintenance of participants’ anonymity during database analyses. No individual consent was obtained.

### Statistical analysis

Data are presented as mean ± standard deviation if normally distributed or as median (interquartile range) if non-normally distributed. Continuous variables were tested with the Kolmogorov–Smirnov test for normal distribution. Categorical variables were given as frequencies and percentages. A chi-squared test or Fisher’s exact test was used for the comparison of categorical variables between groups, and Student’s *t*-test or the Mann–Whitney *U* test was performed for the comparison of continuous variables between the groups. All-cause mortality and freedom from appropriate ICD therapy were calculated using Kaplan–Meier analysis with the procedure serving as the index event. All *p*-values were two-tailed and were considered statistically significant if their value was <0.05. Statistical analyses were performed using SPSS Statistics (IBM, Chicago, IL, USA).

## Results

A total of 53 patients underwent VTA supported by VA-ECMO between January 2014 and December 2020 (Figure 1). Of these, two required VA-ECMO for hemodynamic support regardless of the ablation. Due to hemodynamic collapse during VTA, VA-ECMO was urgently introduced as a bailout strategy in five patients. These patients were excluded from the current analysis. In the remaining 46 consecutive cases (93.5% male, age 62 ± 10 years), VA-ECMO was placed preemptively, specifically to support the ablation procedure, forming the study population. Patients’ clinical characteristics at baseline are shown in Table 1. The majority of patients had ischemic cardiomyopathy were being chronically treated with class III antiarrhythmic drugs on a long-term basis and had VT storm. Half had an NYHA functional class III or IV.

**TABLE 1:** Patient baseline characteristics.

	All (*N* = 46)	Early de-cannulation (*N* = 38)	No early de-cannulation (*N* = 8)	*p*-value
Male	43 (93.5)	35 (92.1)	8 (100)	1
Age	62 ± 10	61.5 ± 10.6	67.6 ± 3	0.005
Hypertension	23 (50)	19 (50)	4 (50)	1
Diabetes mellitus	14 (30.4)	12 (31.6)	2 (25)	1
Chronic kidney disease	9 (19.6)	7 (18.4)	2 (25)	0.645
Atrial fibrillation	17 (37)	12 (31.6)	5 (62.5)	0.125
Ischemic cardiomyopathy	30 (65.2)	25 (65.8)	5 (62.5)	1
Non- Ischemic cardiomyopathy	12 (26.1)	10 (26.3)	2 (25)	1
Chronic obstructive lung diseases	4 (8.7)	4 (10.5)	0	1
Past VT ablation	23 (50)	19 (50)	4 (50)	1
NYHA FC III-IV	23 (50)	18 (47.4)	5 (62.5)	0.699
Decompensated HF	10 (21.7)	7 (18.4)	3 (37.5)	0.344
VT storm	32 (69.6)	25 (65.8)	7 (87.5)	0.403
Presenting arrhythmia				
Monomorphic VT	44 (95.6)	36 (94.7)	8 (100)	1
Polymorphic VT	1 (2.2)	1 (2.6)	0	1
Ventricular fibrillation	1 (2.2)	1 (2.6)	0	1
Hemodynamically unstable VT/VF	24 (52.2)	20 (52.6)	4 (50)	1
Antiarrhythmic drugs				
Amiodarone	32 (65.3)	25 (65.8)	7 (87.5)	0.403
Sotalol	10 (21.7)	10	0	0.171
Baseline vitals				
Heart rate		70.9 ± 18	74 ± 18	0.641
Systolic BP		117 ± 25	108 ± 19	0.358
Diastolic BP		67 ± 15	67 ± 10	0.992
Chronic medical treatment				
Beta blockers	46 (100)	38 (100)	8 (100)	-
ACE/ARB	39 (84.8)	32 (84.2)	7 (87.5)	1
MRA	38 (82.6)	31 (81.6)	7 (82.6)	1
LVEF,%	19.5 ± 8.5	20.3 ± 9	15.6 ± 5	0.053
LVEF < 20%		26 (68.4)	8 (100)	0.09
SPAP		43 ± 17	48 ± 18	0.479
Severe PHTN	6 (15.8)	4 (12.1)	2 (40)	0.169
≥Moderate RV dysfunction,%	13 (28)	9 (24.3)	4 (50)	0.202
PAAINESD score	19.4 ± 7.01	18.8 ± 7.2	22 ± 5.7	0.247

ACE, angiotensin-converting enzyme; ARB, angiotensin II receptor blockers; BP, blood pressure; LVEF, left ventricle ejection fraction; NYHA, New York Heart Association; PHTN, pulmonary hypertension; RV, right ventricle; SPAP, systolic pulmonary arterial pressure; VT, ventricular tachycardia; VF, ventricular fibrillation.

Forty patients underwent early de-cannulation in the electrophysiology lab immediately following ablation. Thirty-eight of these remained free of circulatory support for the remainder of the hospital stay and formed group A – successful early de-cannulation. Group B comprised patients for whom early de-cannulation was deemed impossible (*n* = 6) and two patients who required reestablishment of VA-ECMO support due to progressive hemodynamic compromise within 24 h of the procedure. Group A tended to be younger and showed a trend toward lower left ventricular ejection fraction (LVEF) when compared to patients in group B (Table 1). We found no other statistically significant differences between the groups. Of note, five patients from group B were successfully de-cannulated within 24 h of the ablation. None of those required further circulatory support.

Details for the ablation procedures are shown in Table 2. The left ventricle was accessed *via* retrograde approach in the majority of cases (*N* = 38, 83%) followed by substrate mapping in sinus rhythm in 43 (93%). Epicardial access was performed in 12 (26%) cases. VTs were successfully induced in the vast majority of cases (89%) with a median of two (IQR 1–3) distinct circuits in each case. The induction of multiple VTs (≥3) was not common and observed in almost a third of cases. Entrainment and full activation mapping were performed in six (13%) and 31 (67%) cases, respectively, targeting a median of two (IQR 1–3) circuits. Complete non-inducibility was achieved in 34 (74%) cases, and clinical VT alone was eliminated in 39 (93%). When comparing groups A and B, we found a higher rate of activation mapping in latter (67 vs. 100%, *p* = 0.03), a greater proportion of inducing multiple VTs (24 vs. 64%, *p* = 0.04), and a higher rate of achieving non-inducibility in the former (82 vs. 38%, *p* = 0.02).

**TABLE 2:** Procedural data.

	All (*N* = 46)	Early de-cannulation (*N* = 38)	No early de-cannulation (*N* = 8)	*p*-value
General anesthesia	41 (89.1)	33 (87)	8 (100)	0.569
Access				
Trans septal	4 (8.7)	3 (7.9)	1 (12.5)	
Retrograde	38 (82.6)	32 (69.6)	6 (13)	
Combined access	4 (8.7)	3 (7.9)	1 (12.5)	
Mapping technique				
VT induction (Any)	41 (89.1)	34 (89.5)	8 (100%)	1
VT induction (clinical)	35 (76.1)	28 (73.7)	7 (87.5)	0.658
Pace-mapping	37 (80.4)	29 (76.3)	8 (100)	0.324
Activation mapping	31 (67.4)	23 (60.5)	8 (100)	0.03
Entrainment	6 (13)	4 (10.5)	2 (25)	0.277
Substrate mapping	43 (93.5)	35 (92.1)	8 (100)	1
Ablation strategy				
Endocardial	33 (71.7)	28 (60.9)	5 (62.5)	
Epicardial	2 (4.3)	2 (5.3)	0	
Endocardial + epicardial	10 (21.7)	7 (18.4)	3 (37.5)	
Trans-coronary	1 (2.2)	1 (2.6)	0	
Lactic acid	14.7 ± 11.7	11.9 ± 4	23 ± 24	0.182
Number of VTs induced	2 (1–3)	2 (1–2)	3 (1–7)	0.079
Induction of ≥ 3 VTs	9 (23.7)	5 (62.5)	14 (30.4)	0.044
Number of VTs targeted	2 (1–3)	2 (1–2)	2 (1–3)	0.416
Procedure duration, min	280 (227–335)	385 (236–405)	255 (221–283)	0.147
Acute outcomes				
Elimination of clinical VT	39 (92.8)	33 (86.8)	6 (75)	0.587
Non-inducibility	34 (74)	31 (81.6)	3 (37.5)	0.02
Inducibility not tested	6 (13)	4 (10.5)	2 (25)	1
Failure	1 (2.2)	1 (2.6)	0	1

VT, ventricular tachycardia.

Less inducible VTs and non-inducibility at the end of the procedure were the only predictors of successful early de-cannulation (1.45 [1.02–2.06], *p* = 0.037 and 0.13 [0.03–0.70], *p* = 0.018, respectively). Failure of immediate de-cannulation was predicted by the induction of multiple distinct VTs with a 45% increased risk of failure for each additional circuit (OR = 1.45 95% CI = 1.02–2.06; *p* = 0.037). Additionally, a high lactic acid level (≥20 mg/dl) close to the end of the procedure showed a trend toward higher risk of early weaning failure (Table 3).

**TABLE 3:** Predictors of failed early de-cannulations.

	OR (95% CI)	*p*-value
LVEF,%	0.913 (0.81–1.03)	0.155
**Non-inducibility**	**0.135 (0.03–0.70)**	**0.018**
Age	1.08 (0.98–1.19)	0.127
PAAINESD	1.07 (0.95–1.21)	0.246
**Number of VT induced**	**1.45 (1.02–2.06)**	**0.037**
**Induction of ≥ 3 VTs**	**5.4 (1.07–27)**	**0.041**
Lactic acid ≥ 20 mg/dl	6.6 (0.86–50.541)	0.069

LVEF, left ventricle ejection fraction; VT, ventricular tachycardia. Bold indicates statistically segnificnt findings.

### Short-term outcomes

A total of nine (19.6%) had major adverse outcomes (Table 4). A single patient sustained a stroke and two developed vascular complications requiring surgical intervention (pseudo-aneurysm and thrombotic occlusion of a femoral artery). In addition, one patient developed intractable cardiogenic shock and died despite continued VA-ECMO support. Another was initially weaned from VA-ECMO but deteriorated within 24 h and was urgently reconnected to VA-ECMO. He underwent implantation of a left ventricular assist device (LVAD, HeartMate 3™, Abbott Laboratories, Chicago, IL, USA) within 6 days. He developed acute right ventricular failure soon thereafter and a right ventricular assist device was implanted (Levitronix) but he died within a few days. A sixth patient was weaned off the VA-ECMO but died from septic shock due to hospital-acquired pneumonia. A seventh patient was kept off VA-ECMO support until he was implanted with LVAD (HeartMate 3™) but died within 24 h of the procedure, 26 days after the ablation. Finally, two patients developed cardiogenic shock that was successfully managed and they recovered. Overall, in-hospital mortality was higher among patients that were not successfully weened of ECMO in the EP lab (5 vs. 50%, *p* = 0.005).

**TABLE 4:** Complications.

	Early de-cannulation (*N* = 38)	No early de-cannulation (*N* = 8)	*p*-value
Vascular complication	2 (5.3)	0	1
CVA	1 (2.6)	0	1
**Cardiogenic shock**	**3 (7.9)**	**4 (50)**	**0.012**
Tamponade	0	0	NA
**In-hospital mortality**	**2 (5.2)**	**4 (50%)**	**0.005**
**Any major complication^#^**	**5 (13.2)**	**4 (50)**	**0.017**

CVA, cerebrovascular accident. ^#^Major complication includes CVA, vascular complication requiring intervention or new cardiogenic shock, cardiac tamponade, and in-hospital death. Bold indicates statistically segnificnt findings.

### Post-discharge outcomes

**FIGURE 2:**
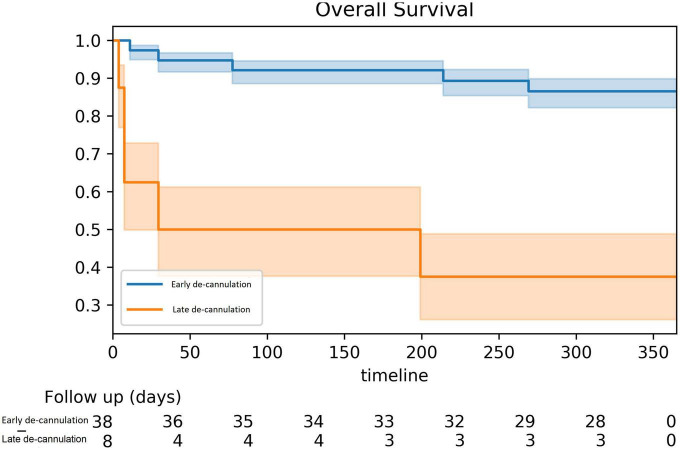
Kaplan–Meier survival analysis in early vs. late de-cannulation.

Over a median follow-up of 15.9 (6.9–31.3) months, a total of 15 (32.6%) patients died. All-cause mortality at 1 year was higher among patients that failed immediate de-cannulation 86 vs. 38% at 1 year (log-rank *p* < 0.001, Figure 2). At 1 year of follow-up, 91.3% of surviving patients were free of appropriate ICD shocks and VT.

## Discussion

This study adds to the accumulating experience of preemptive use of VA-ECMO to support VT ablation in high-risk patients and presents, for the first time, the clinical impact of routine early de-cannulation at the end of the procedure. Early de-cannulation was performed successfully in the majority (82.6%) of cases despite the relatively high-risk profile of our cohort. This was predicted by a lower number of inducible VTs and by achieving non-inducibility at the end of the ablation. Failure to wean off VA-ECMO support was associated with multiple distinct VTs circuits induced and by high lactic acid levels, measured close to the end of the procedure. Furthermore, failure to wean off VA-ECMO, prolonged VA-ECMO support, or urgent re-cannulation following an apparently successful weaning process was all associated with excess mortality. This highlights the critical importance of the early post-procedural course as a powerful predictor of poor outcomes and a possible trigger for urgent intervention.

Drug refractory VTs in the presence of structurally abnormal hearts pose a high stake therapeutic challenge. Often, VTA is the only strategy that may offer long-term freedom for arrhythmia. In recent years, it became clear that the safe and effective execution of these procedures requires careful risk stratification and preparation. Acute hemodynamic collapse during the ablation has been associated with extremely high mortality despite rescue attempts with circulatory support. Importantly, this was frequently observed during sinus rhythm or pace mapping. Therefore, hemodynamic collapse may not be avoided by limiting the procedure to a substrate base approach. As a result, the preemptive use of circulatory support systems during VTA emerges as an important approach for selected high-risk patients. The PAINESD score has gained acceptance as a useful risk stratification scheme (12) developed to predict hemodynamic collapse during VTA and the need for the preemptive use of circulatory support. As described above, we have used a different set of criteria for patient selection, but the PAINESD score of our patients is comparable to previous publications, allowing for comparisons with previous cohorts.

Veno-arterial extracorporeal membrane oxygenation is used, typically, in acute settings of typical providing support for a few days to a few weeks, depending on the clinical scenario and patient condition. It is used both as a bridge for recovery and a bridge for a different definitive treatment or until the patient dies. Increased experience and the growing number of specialist centers registered with ELSO have led to increased use of VA-VA-ECMO in more elective cases, such as in high-risk percutaneous coronary intervention, high-risk percutaneous structural heart procedures, and is being used as a “stand-by” for different high-risk procedures.

Published reports of VTA with hemodynamic support also describe the use of Impella ventricular support device and/or TandemHeart. Nevertheless, VA-ECMO has the potential for more complete hemodynamic support (up to 5 l/min) as well as respiratory support. Furthermore, the system does not affect the access to the LV as the Impella and may be used in the presence of the mechanical aortic valve.

Importantly, in previous reports, patients were kept on circulatory support for at least 24 h after the procedure (7). The utilization of VA-ECMO is associated with a risk of significant complications. While those have been shown to be well balanced by the benefit of intra-procedural support and the prevention of hemodynamic collapse, more prolonged use must be further justified. The use of VA-ECMO mandates continued anticoagulation resulting in an ongoing risk of major bleeding, particularly at the cannula insertion site. Similarly, the risk of nosocomial infection (13) and limb ischemia increases dramatically as a function of the duration of therapy. Lastly, maintaining VA-ECMO support takes a significant toll on hospital’s limited resources. Therefore, minimizing the exposure to VA-ECMO is a priority when possible. Our results show that immediate de-cannulation is feasible and safe in the majority of cases. Therefore, we propose that a careful weaning process at the end of VTA, particularly when successful, should be attempted in most cases. Our results are consistent with a recent study by Muser et al. reporting a similar rate of 96% immediate removal of circulatory support at the end of the procedure (4). Notably, in that cohort, the Impella 2.5 or Impella CP pLVAD devices (Abiomed, Inc., Danvers, MA, USA) were used, not the VA-ECMO (4).

Despite our previous statement, premature weaning may be detrimental. Two cases in which immediate cannulation was performed, followed by the reinstitution of VA-ECMO support soon thereafter, resulted in death within 30 days. This happened despite the implantation of an LVAD system in one of the patients. It is possible that uninterrupted VA-ECMO support as a bridge to LVAD would have resulted in a better outcome. In our cohort, the indication of multiple VTs, probably as a maker of advanced cardiomyopathy with the extensive electrical substrate, emerged as a sign of poor outcome and should merit caution. Additional risk factors that approached statistical significance were advanced age and very low LVEF (<20%).

It is probable that the cumulative time a patient spent in VT may have a substantial impact on his hemodynamic stability even with ECMO support. While the rate of activation mapping was higher in the late cannulation group, it did not result in a longer overall procedural duration and it was not a predictor of failed early de-cannulation. We were unable to report the total time spent in VT during the procedure or the longest VT episode and therefore could not draw specific conclusions on the importance of limiting the duration of activation mapping. Nevertheless, based on our early clinical experience, our current practice is to limit the duration of mapping for a single VT circuit to a maximum of 30 mins.

Lastly, while ECMO may allow comprehensive activation mapping and safer execution of post-ablation programmed stimulation protocols, circulatory support should not be regarded as a means to improve acute success (non-inducibility) in most VTAs. Rather, it should be regarded as a tool that enables the safe completion of VTAs in very high-risk patients. The rates of non-inducibility achieved in our cohort are comparable to several recent VTA studies but are by no means superior to them. Yet the patients comprising our cohort were substantially sicker, with lower LVEFs, more advanced HF, a higher proportion of VT storm, and previous failed ablations and we perfumed aggressive program stimulation protocols (up to 3 and even 4 extra-stimuli) as a routine. Therefore, the use of ECMO support may enable a high-risk patient to complete a comprehensive and rigorous VTA that would otherwise be impossible.

### Limitations

We acknowledge several limitations. Mainly the retrospective nature of this analysis of a single-center experience. Furthermore, the study’s sample size precluded extensive statistical analysis. Yet, our cohort is very similar to previously published reports allowing generalization of our results to other high-risk patients with structurally abnormal hearts intended for ECMO-supported VTA. Moreover, the question of immediate early de-cannulation is unlikely to be tested in a randomized prospective fashion emphasizing the importance of reports like this one. Our pre-procedural evaluation protocol did not include advanced imaging aimed at identifying the arrhythmogenic substrate. The use of multimodality computed tomography and particularly cardiac magnetic resonance, when clinically feasible, may have reduced mapping and ablation time, thus contributing to the safety of the procedures (14, 15). Lastly, the study was not designed to assess the benefit of ECMO support for VT ablation in high-risk patients.

## Conclusion

Immediate de-cannulation from VA-ECMO support at the end of VT ablation is feasible in the majority of cases. Close hemodynamic monitoring and a careful weaning process are warranted. The likelihood of success is high when complete non-inducibility was achieved and lower when multiple VT circuits were induced. Further study is needed to better define the predictors of successful immediate de-cannulation.

## Data availability statement

The raw data supporting the conclusions of this article will be made available by the authors, without undue reservation.

## Ethics statement

The studies involving human participants were reviewed and approved by Sheba Medical Center’s Ethical Board. Due to the retrospective nature of the analysis, no individual consent was required. Written informed consent for participation was not required for this study in accordance with the national legislation and the institutional requirements.

## Author contributions

AS, RB, and EN contributed to conception and design of the study. JN and EN organized the database. AS and JN performed the statistical analysis. AS and EN wrote the first draft of the manuscript. RB, LS, IK, AK, and ER wrote sections of the manuscript. All authors contributed to manuscript revision, read, and approved the submitted version.

## Abbreviations

CL: cycle length
LVAD: left ventricular assist device
LVEF: left ventricular ejection fraction
SMVT: sustained monomorphic ventricular tachycardia
VA-ECMO: veno-arterial extracorporeal membrane oxygenation
VT: ventricular tachycardia
VTA: ventricular tachycardia ablation.
