# Supplementary material for: Early de-cannulation from extracorporeal membrane oxygenation following ventricular tachycardia radiofrequency ablation
Source: Front Cardiovasc Med. 2022 Oct 18;9:998079. doi: 10.3389/fcvm.2022.998079 (PMC9622793; doi:10.3389/fcvm.2022.998079)
Supplement: Supplementary file 1 [file Table_1.DOCX]

**Supplementary table – eligibility of ECMO supported VT ablation**

| Patient number | Severe AS | Severe PTH | Decompensated heart failure | Severe HF | Hemodynamically unstable VT | Previously failed VT ablation | Idiopathic dilated cardiomyopathy | Eligibility |
| --- | --- | --- | --- | --- | --- | --- | --- | --- |
| 1 | 0 | 0 | 0 | 0 | 1 | 1 | 1 | Yes |
| 2 | 0 | 0 | 0 | 1 | 0 | 1 | 0 | Yes |
| 3 | 0 |  | 0 | 1 | 1 | 0 | 0 | Yes |
| 4 | 0 | 0 | 1 | 1 | 0 | 1 | 0 | Yes |
| 5 | 0 | 0 | 1 | 1 | 1 | 0 | 0 | Yes |
| 6 | 0 | 0 | 1 | 0 | 0 | 1 | 1 | Yes |
| 7 | 0 |  | 0 | 0 | 0 | 0 | 0 | No |
| 8 | 0 | 0 | 0 | 1 | 1 | 1 | 1 | Yes |
| 9 | 0 | 0 | 0 | 0 | 0 | 1 | 1 | Yes |
| 10 | 0 |  | 0 | 1 | 0 | 1 | 0 | Yes |
| 11 | 0 | 0 | 0 | 1 | 0 | 1 | 1 | Yes |
| 12 | 0 | 0 | 0 | 0 | 0 | 1 | 1 | Yes |
| 13 | 0 | 0 | 0 | 1 | 1 | 0 | 0 | Yes |
| 14 | 0 | 0 | 1 | 1 | 1 | 0 | 0 | Yes |
| 15 | 0 |  | 0 | 1 | 1 | 0 | 1 | Yes |
| 16 | 0 |  | 0 | 0 | 0 | 0 | 1 | Yes |
| 17 | 0 | 0 | 0 | 0 | 0 | 1 | 0 | Yes |
| 18 | 0 | 1 | 1 | 0 | 1 | 0 | 0 | Yes |
| 19 | 0 | 0 | 0 | 0 | 0 | 0 | 1 | Yes |
| 20 | 0 | 0 | 0 | 1 | 0 | 0 | 0 | Yes |
| 21 | 0 | 0 | 0 | 0 | 1 | 1 | 1 | Yes |
| 22 | 0 |  | 0 | 0 | 1 | 0 | 0 | Yes |
| 23 | 0 | 0 | 0 | 1 | 1 | 0 | 0 | Yes |
| 24 | 0 | 0 | 0 | 0 | 0 | 1 | 0 | Yes |
| 25 | 0 | 0 | 0 | 0 | 0 | 1 | 0 | Yes |
| 26 | 0 | 0 | 0 | 1 | 0 | 1 | 0 | Yes |
| 27 | 0 | 1 | 0 | 0 | 1 | 0 | 0 | Yes |
| 28 | 0 | 0 | 0 | 0 | 1 | 0 | 0 | Yes |
| 29 | 0 | 0 | 0 | 0 | 0 | 1 | 0 | Yes |
| 30 | 0 | 0 | 0 | 1 | 1 | 0 | 0 | Yes |
| 31 | 0 | 0 | 0 | 1 | 0 | 0 | 0 | Yes |
| 32 | 0 | 0 | 0 | 1 | 1 | 1 | 0 | Yes |
| 33 | 0 | 1 | 1 | 0 | 1 | 1 | 0 | Yes |
| 34 | 0 |  | 1 | 1 | 1 | 1 | 0 | Yes |
| 35 | 0 | 0 | 1 | 0 | 1 | 0 | 0 | Yes |
| 36 | 0 | 0 | 0 | 0 | 1 | 1 | 0 | Yes |
| 37 | 0 | 1 | 0 | 0 | 1 | 1 | 0 | Yes |
| 38 | 0 | 1 | 1 | 0 | 0 | 0 | 0 | Yes |
| 39 | 0 | 0 | 0 | 1 | 0 | 1 | 0 | Yes |
| 40 | 0 | 0 | 0 | 1 | 1 | 1 | 0 | Yes |
| 41 | 0 |  | 0 | 1 | 0 | 1 | 0 | Yes |
| 42 | 0 | 0 | 0 | 1 | 1 | 0 | 0 | Yes |
| 43 | 0 | 1 | 1 | 1 | 1 | 0 | 0 | Yes |
| 44 | 0 | 0 | 0 | 1 | 0 | 0 | 0 | Yes |
| 45 | 0 | 0 | 0 | 0 | 0 | 0 | 1 | Yes |
| 46 | 0 | 0 | 0 | 0 | 1 | 1 | 1 | Yes |
